# Analysis of Alpha-Synuclein in Malignant Melanoma – Development of a SRM Quantification Assay

**DOI:** 10.1371/journal.pone.0110804

**Published:** 2014-10-21

**Authors:** Charlotte Welinder, Göran B. Jönsson, Christian Ingvar, Lotta Lundgren, Bo Baldetorp, Håkan Olsson, Thomas Breslin, Melinda Rezeli, Bo Jansson, Thomas E. Fehniger, Thomas Laurell, Elisabet Wieslander, Krzysztof Pawlowski, György Marko-Varga

**Affiliations:** 1 Division of Oncology and Pathology, Clinical Sciences, Lund University, Lund, Sweden; 2 Centre of Excellence in Biological and Medical Mass Spectrometry, Lund University, Lund, Sweden; 3 Skåne University Hospital, Lund, Sweden; 4 Dept. of Surgery, Clinical Sciences, Lund University, Skåne University Hospital, Lund, Sweden; 5 Dept. of Cancer Epidemiology, Clinical Sciences, Lund University, Lund, Sweden; 6 Clinical Protein Science & Imaging, Biomedical Center, Biomedical Engineering, Lund University, Lund, Sweden; 7 Dept. of Experimental Design and Bioinformatics, Faculty of Agriculture and Biology, Warsaw University of Life Sciences, Warszawa, Poland; 8 First Department of Surgery, Tokyo Medical University, Tokyo, Japan; Cellcuity, United States of America

## Abstract

Globally, malignant melanoma shows a steady increase in the incidence among cancer diseases. Malignant melanoma represents a cancer type where currently no biomarker or diagnostics is available to identify disease stage, progression of disease or personalized medicine treatment. The aim of this study was to assess the tissue expression of alpha-synuclein, a protein implicated in several disease processes, in metastatic tissues from malignant melanoma patients. A targeted Selected Reaction Monitoring (SRM) assay was developed and utilized together with stable isotope labeling for the relative quantification of two target peptides of alpha-synuclein. Analysis of alpha-synuclein protein was then performed in ten metastatic tissue samples from the Lund Melanoma Biobank. The calibration curve using peak area ratio (heavy/light) versus concentration ratios showed linear regression over three orders of magnitude, for both of the selected target peptide sequences. In support of the measurements of specific protein expression levels, we also observed significant correlation between the protein and mRNA levels of alpha-synuclein in these tissues. Investigating levels of tissue alpha-synuclein may add novel aspect to biomarker development in melanoma, help to understand disease mechanisms and ultimately contribute to discriminate melanoma patients with different prognosis.

## Introduction

*The latest epidemiological statistics position malignant melanoma (MM)* as the third and most deadly type of skin cancer, while basal cell carcinoma is by far the most common type of skin cancer. Malignant melanoma develops in melanocytes, i.e., the pigment producing cells in the skin. Although MM accounts for only 4% of all skin cancers, it is more aggressive than the other types of skin cancer, and accounts for 80% of the mortality related to skin cancer [1].

Alpha-synuclein, encoded by the SNCA gene, is a protein with a yet unknown but complex mechanism of action in diseases. Alpha-synuclein is a synuclein protein with multiple functions, such as being involved in mitochondrial dysfunction, nuclear localization, vesicle trafficking etc [2]. Insights on altered mitochondrial function and dynamics in the pathogenesis of neurodegeneration [2]–[4] may help understand the role of alpha-synuclein in Parkinson’s disease (PD). Alpha-synuclein is primarily found in neural tissue making up as much as 1% of all proteins in the cytosol, but also in melanoma and nevus tissues [5]. Mutation in the alpha-synuclein gene (SNCA) as well as misfolding, and accumulation of the protein have been implicated in the development of PD [4], [6]. Recently, Matsuo et al have shown that determination of alpha-synuclein protein expression could be useful also for the diagnosis of metastatic melanoma, although it cannot be used to distinguish between malignant and benign melanocytic skin lesions since melanosomes express alpha-synuclein [7]. In melanocytic cells, the protein expression of alpha-synuclein may be regulated by microphthalmia-associated transcription factor (MITF) [8]. MITF is a master regulator gene of melanocyte development and differentiation and is also associated with melanoma development and progression [9], [10]. Lately, there has been growing evidence in the literature for mutual mechanisms between cancer and CNS disorders [11], and especially on shared risk and overlapping disease mechanisms in the development of PD and MM [12]–[15]. These findings suggested a link between MM and PD. Within the disease pathology of PD, alpha-synuclein is involved in a major pathway for protein aggregation. The monomeric protein form is natively unfolded, but will bind to membranes in an α-helical form. From this, unfolded monomers will aggregate first into small oligomeric species that can be stabilized by β-sheet interactions, and then into higher molecular weight insoluble fibrils [16]. Interaction with lipids is one of the ways aggregation occurs and promotes oligomer formation. The deposition of alpha-synuclein into pathological structures such as Lewy bodies is probably a late event that occurs and causes toxicity in neurons and neuronal cell death. Current hypotheses focus towards alpha-synuclein oligomers being the more toxic species [4], [17], [18].

The causal link between melanoma and PD may center on tyrosine metabolism [19]. Alpha-synuclein has been shown to negatively regulate the activity of tyrosine hydroxylase [20], [21], the rate-limiting enzyme in the production of dopamine and melanin [22]. Additional data provide supporting evidence for the existence of a common, or at least related, pathogenic disease mechanism between MM and PD [13], [23]. The interaction between alpha-synuclein and tyrosinase may occur more frequently in patients with PD who have shortage in dopamine levels, and the fibrillar forms of alpha-synuclein within PD disease may be responsible for impairments within the tyrosine pathway involved in melanogenesis, predisposing the individual to melanoma [19]. Notably however, there seem to be no direct positive correlation between melanin and alpha-synuclein expression in melanoma tissue cells. Thus, more than fifty percent of the cells highly expressing alpha-synuclein in melanoma were lacking melanin pigments [7].

Quantification of alpha-synuclein in human cerebrospinal fluid (CSF) has been suggested to serve as a biomarker candidate for PD [24]. However, recent works from several groups trying to quantify alpha-synuclein is inconsistent with the reported absolute concentrations of alpha-synuclein. Some studies found reduced concentration in CSF in PD relative to controls [25]–[29] and another study reported no change [30].

There are at least four isoforms of alpha-synuclein, which are produced through alternative splicing. The major form of the protein is the full length form with a 140 amino acids long transcript. The other isoforms are alpha-synuclein-126, where the exon 3 is lost and the protein lacks residues 41–54. The alpha-synuclein-112 lacks residues 103–130 due to loss of exon 5 [31]. Finally, alpha-synuclein-98 lacks exon 3 and 5. Alpha-synuclein structure also goes through post-translational modifications in a number of annotations, as well as alternative splicing as aggregation enhancers [31], [32]. In addition, truncated forms as well as peptide cleavage products of alpha-synuclein has been shown to have chemotactic functions, in addition to a number of complexes occurring with other target proteins [33]–[38]. These highly complex and potentially disease driven alterations of alpha-synuclein makes it an enormous challenge trying to capture the full profile related to MM and to elucidate the various peptide functions. This includes what detailed mechanisms drug molecule(s) needs to be directed towards in order to reach efficacy, using conventional immunoassay technologies.

By using novel mass spectrometry technology a SRM assay was developed and validated in order to capture the interplay of alpha-synuclein peptides within MM disease mechanisms, thus being able to quantify the entire cascade of variants by nano LC separation interfaced to tandem mass spectrometry.

The expression level of alpha-synuclein was evaluated within metastatic tissue samples from patients diagnosed with stage III melanoma. The mRNA levels of the gene SNCA was analyzed by microarray in the same metastatic tissue samples.

## Materials and Methods

### 2.1. Clinical Samples

Ten lymph node metastasis samples (Stage III) from MM cancer patients, archived in the local malignant melanoma biobank were obtained from Skåne University Hospital, Sweden. The clinical information on respective patients is summarized in Table 1. Ethical approval was granted by Central Ethical Review board at Lund University; approval number: DNR 191/2007, 101/2013. All patients within the study provided a written informed consent. The malignant melanoma biobank is located at Barngatan 2B, 221 85 Lund, Sweden. The biobank is called “Tissuebank for research on tumor diseases” (BD20).

**Table 1:** Clinical information of patient characteristics. Breslow thickness and Clarks refer to primary melanoma feature.

Tumor	Gender	Age at metastases	Age at primary	Breslow class	Clark	Stage	Status
MM35	Male	55	54	3	4	3	Alive
MM98	Male	75	73	4	4	3	Dead
MM504	Male	54	NA	NA	NA	NA	Dead
MM687	Male	74	72	1	2	3	Dead
MM787	Male	81	78	2	4	3	Dead
MM812	Male	51	NA	NA	NA	NA	Alive
MM813	Female	54	54	2	3	3	Alive
MM825	Female	66	64	2	4	3	Alive
MM829	Male	55	49	1	2	3	Alive
MM835	Female	36	32	3	3	3	Alive

NA-not available.

### 2.2. Sample Preparation

Proteins and mRNAs were extracted from frozen melanoma tumor tissue. Tissues were carefully dissected during surgery and subdivided into 5–8 mm^2^ fragments and placed into cryo-tubes. Tissue (15–20 mg), were processed by TissueLyser (Qiagen, Hilden, Germany) and AllPrep DNA/RNA/Protein Mini Kit (Qiagen Ltd, Crawley; UK), according to the manufacturer's’-instructions. Extracted proteins were precipitated with ice-cold acetone to a final concentration of 80% acetone and incubated for 30 min at −20 C followed by centrifugation at 16000 g for 2 minutes. The supernatant was removed, and the protein pellets were allowed to air dry. The dried protein pellets were resolved in 8 M urea in 50 mM ammonium bicarbonate (pH 7.6). Protein concentration was determined by the BCA method (Pierce, Rockford, IL, USA). From the total protein fraction, 150 µg, was reduced with 10 mM DDT (1 h at 37 C) and alkylated using 40 mM iodoacetamide (30 min, kept dark at room temperature). Buffer were exchanged to 50 mM ammonium bicarbonate buffer (pH 7.6) using a 10 kDa cut-off spin filter (YM10 filter, AMICON). The samples were subsequently digested with sequencing grade trypsin (Promega, Madison, WI, USA) overnight at 37°C with a of ratio 1∶120 w/w (trypsin:protein).

### 2.3. mRNA Analysis

SNCA mRNA levels for the ten melanoma tumors analyzed by the SRM assays were extracted from a whole genome gene expression assay (Illumina CA) on HT-12 v4 arrays (Cirenajwis et al. in preparation).

### 2.4 Haemoglobin Analysis – Western Blot

The ten tumor lysates, 20 µg, was diluted in NuPAGE LDS sample buffer (Invitrogen, California, US) with 50 mmol/L dithiotreitol (DTT) and incubated at 95°C for 10 min. Thirty µg of the protein was separated using 4–12% NuPAGE, Bis-Tris, 1 mm thick gels with 15 wells (Invitrogen, California, US) with SeeBlue Plus2 (Invitrogen, California, US) as molecular mass standard. Whole blood from a healthy volunteer (female) was used as a positive control for the rabbit anti-human haemoglobin (A0118, Dako). The electrophoresis was run in MES buffer at 180 V for 1 h and the proteins were then transferred to 0.2 µm PVDF membrane (Trans-Blot Turbo Transfer Pack, Mini format, Bio-Rad) at 25V for 30 min using Trans-Blot Turbo Transfer System (Bio-Rad). The membrane was blocked in 5% non-fat dry milk in 0.2% Tween-20, 150 mM NaCl and 20 mM Tris, pH 7.5 (TTBS) for 3 hours and incubated with rabbit anti-human haemoglobin (10 µg/mL) over night. The membrane was washed three times in TTBS, 10 min each. The membrane was incubated with FITC conjugated polyclonal swine anti-rabbit Immunoglobulins diluted 1∶50, for 1 hour, washed three times, 10 min each, with TTBS and antibody binding was detected with Gel Doc EZ Imager (Bio-Rad). The staining density for each band was analysed with Image Lab Software (Bio-Rad).

### 2.5. In Silico Selection of Signature Peptides

The theoretical digestion of the neXtProt entry NX_P37840 was performed by the PeptideMass tool (available at the ExPASy Proteomics Server website, http://expasy.org/sprot/ [39] using the following settings: iodoacetamide as alkylation agent with oxidation on methionine and no miss-cleavage. The resulted tryptic peptides were investigated for uniqueness by using Basic Local Alignment Search Tool (BLAST) [40]. Finally, two tryptic peptides were chosen for crude peptide synthesis with and without heavy isotope labeling.

### 2.6. SRM Assay Development

Crude peptides, both light and heavy, were supplied by Thermo Scientific (Ulm, Germany). The heavy peptides were isotopically labeled on the C-terminal-lysine residue (^13^C, ^15^N). A mixture was created for the two peptides (EQVTNVGGAVVTGVTAVAQK and TVEGAGSIAAATGFVK), corresponding to residues 61–80 and 81–96 of alpha-synuclein, respectively. Based on the total peptide content of the crude peptides, each peptide was diluted to an estimated concentration of 50 fmol/µL. The transition lists were created in Skyline v1.2 software [41] (MacCoss Lab Software, Seattle, WA). Primarily, high numbers of transitions, all possible y-ion series that matches the criteria (from *m/z* >precursor-2 to last ion-2, precursor *m/z* exclusion window: 20 Th), were selected for each peptide at both 2+ and 3+ charge states. The peptide mixture was analyzed by nano LC-MS/MS using a TSQ Vantage triple quadrupole mass spectrometer equipped with an Easy n-LC II pump (Thermo Scientific, Waltham, MA). The samples were injected onto an Easy C18-A1 pre-column (2 cm, ID 100 µm with 5 µm particles) (Thermo Scientific, Waltham, MA), and following on-line desalting and concentration the tryptic peptides were separated on a 75 µm ×150 mm fused silica column packed with ReproSil C18 (3 µm, 120 Å from Dr. Maisch GmbH, Germany). Separations were performed in a 45-min linear gradient from 10 to 35% acetonitrile containing 0.1% formic acid; at a flow rate 300 nL/min. The MS analysis was conducted in positive ion mode with the spray voltage and declustering potential were set to 1750 V and 0, respectively. The transfer capillary temperature was set to 270°C and the amplitude of the S-lens was 143. SRM transitions were acquired in Q1 and Q3 operated at unit resolution (0.7 FWHM), the collision gas pressure in Q2 was set to 1.2 mTorr. The cycle time was 2.5 s and the dwell times were 0.11 and 0.10 for TVEGAGSIAAATGFVK and EQVTNVGGAVVTGVTAVAQK, respectively. The three best transitions per precursor were selected by manual inspection of the data in Skyline and scheduled transition lists were created for the final assays. Collision energies were optimized for each peptide. The collision energy was ramped round the predicted value in 3 steps on both sides, in 2V increments. The optimized collision energies were 21 and 29 for TVEGAGSIAAATGFVK and EQVTNVGGAVVTGVTAVAQK, respectively. The selected transitions were tested in real matrix also by spiking the heavy peptide mixtures into human MM tissue digests.

### 2.7. Standard Curves of Synthetic Peptides

A reverse standard curve approach was used for the calibration curve, thereby providing relative quantitative data to the melanoma patient samples [42]. A dilution series of heavy labeled synthetic peptide mixtures (in the estimated concentration range of 6.25 −200 fmol/µl) in tumor digest containing constant amount of non-labeled synthetic peptides (in the estimated concentration of 100 fmol/µl) for characterization of the assay linearity were analyzed in triplicates. Calibration curves were generated by linear regression analysis on the peak areas ratios (heavy/light) versus concentration ratios for the targeted peptides.

### 2.8. SRM Assay of Alpha-Synuclein

For relative quantification, the two heavy labeled peptides were spiked into ten tumor lysate digests at an estimated concentration of 6.25 fmol/µL and 50 fmol/µL for TVEGAGSIAAATGFVK and EQVTNVGGAVVTGVTAVAQK, respectively.

### 2.9. Data Analysis

Data sets were imported into Skyline (v1.2 http://proteome.gs.washington.edu/software/skyline) and peaks were automatically integrated. After automatic integration of the data sets, the data was also manually inspected. Integration of the peaks was adjusted when signals were not intense and the software could not reliably determine the peak. Interferences with the matrix, detector saturations and variable peak area ratios in replicate samples were also investigated. Data from the individual tumor lysates are presented as mean of triplicates measurements +/− standard deviation. Because originally mRNA expression index was reported after log2 transformation, for correlating mRNA and protein levels the mRNA expression index was transformed by raising 2 to the power equal to the expression index. Pearson correlation between mRNA and proteins levels was then calculated.

## Results

In order to utilize genes and proteins as biomarkers for disease and/or treatments as for drug responders, more experience and Research & Development inputs are requested. For diagnostic quantification of protein(s) in clinical studies, there are currently no standard guidelines from the Food and Drug Administration (FDA) or European Medicines Agency (EMEA) that need to be met for approval. However, there are on-going projects concerning such biomarkers development between the FDA, academics projects and pharmaceutical industry that are investigating standardization procedures for future utilization. A guideline for industry has recently been introduced (http://www.fda.gov/downloads/RegulatoryInformation/Guidances/ucm126957.pdf). The initial step of a SRM assay development usually relates to apply an *in silico* step, where a selection of suitable peptides from the proteins are made, followed by BLAST searching in protein database, where identified peptides from the proteins can verify the utility of target peptides identified as candidates. Precursors and m/z of the peptide products are the keys to the assay development. Recently, a rapid assay development with SRM-MS instrumentations was presented [43]. In this respect, peptides libraries used as standards, are the most valuable tools in order to funnel the large number of peptide candidates in the in silico processing step that make judgments of the most useful target peptide candidates for the assay [44], [45].

### 3.1. Selection of Transitions for SRM

**Figure 1:**
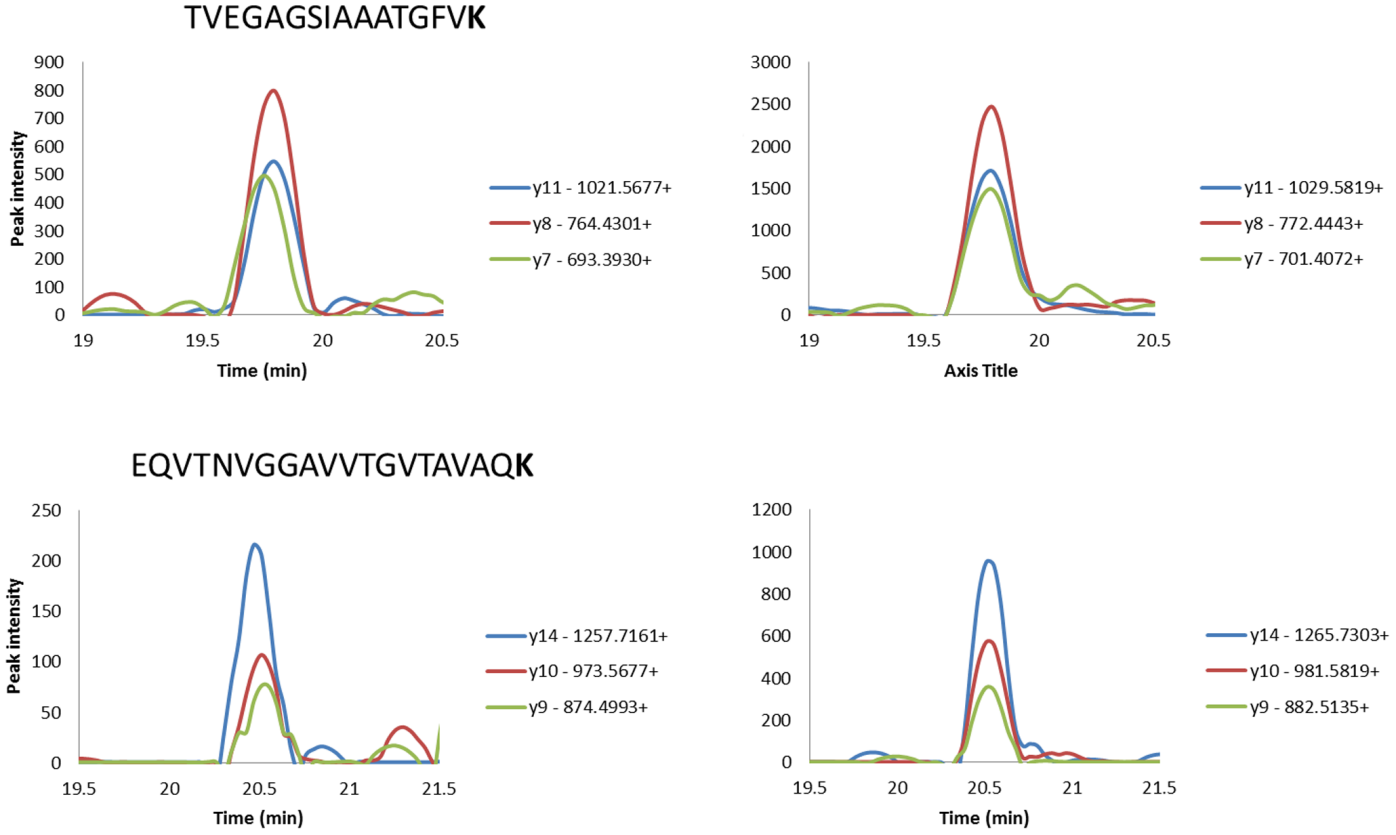
Extracted ion chromatograms show the 3 monitored transitions for the endogenous (left) and the heavy labeled peptides (right) for the two peptides.

By selection of the consensus sequence of alpha-synuclein (neXtProt entry NX_P37840) a theoretical tryptic peptide list was generated. Two sequences were identified; TVEGAGSIAAATGFVK and EQVTNVGGAVVTGVTAVAQK. These peptides were chosen in order to encompass an assay that will provide quantitative data on all three alpha-synuclein isoforms. The peptides were evaluated for their uniqueness using BLAST [40]. The selected peptides were synthesized in both heavy labeled and unlabeled forms in order to determine optimal SRM transitions detected by mass spectrometry. The three highest intensity fragment ions were selected for SRM transitions for each peptide (Table 2). Spiking heavy labeled peptides into the tumor lysate digest made experimental verification of the selected transitions. The relative signal intensities of the daughter ions generated from endogenous peptides were compared to those of the heavy labeled peptides. These experiments verified that the pattern of the daughter ions from the endogenous, and the heavy labeled peptides were the same. This experimental proof is shown in Figure 1, which ensures that the selected transitions are free from interference from matrix.

**Table 2:** Proteotypic peptides sequences and selected SRM transitions for the two peptides.

Accession no	Protein	Position	Peptide sequence	Q1	Q3
**P37840**	a- synuclein	81–96	TVEGAGSIAAATGFVK	739.9 (2+)	1021.6 (y11^1+^)
					764.4 (y8^1+^)
					693.4.4 (y7^1+^)
			TVEGAGSIAAATGFVK	743.9(2+)	1029.6 (y11^1+^)
					772.4 (y8^1+^)
					701.4 (y7^1+^)
		61–80	EQVTNVGGAVVTGVTAVAQK	964.5(2+)	1257.7 (y14^1+^)
					973.6 (y10^1+^)
					874.5 (y9^1+^)
			EQVTNVGGAVVTGVTAVAQK	968.5 (2+)	1265.7 (y14^1+^)
					981.6 (y10^1+^)
					882.5 (y9^1+^)

### 3.2. Quantification and Analytical Assay Performance

Clinical guidelines for creating MS-based assays require that heavy labeled synthetic peptides be added to samples at concentrations close to the mean concentration of the endogenous peptides to permit reproducible measurements [46]. The linearity and reproducibility of the SRM assay was investigated in 6 dilution steps using nano-LC separation. The tissue samples were spiked with different amount of heavy labeled synthetic peptides while keeping amounts of non-labeled synthetic peptides constant. The nano-LC provides improved sensitivity with an acceptable robustness, typically providing a relative standard deviation (RSD) <1% for the retention times.

The chromatographic separation conditions with the Internal Standards (IS), were chosen so that sufficient separation was achieved by the two IS (0.7 min peak-to-peak separation), and at the same time to have a fast turnaround in the cycle time (45 min).

**Figure 2:**
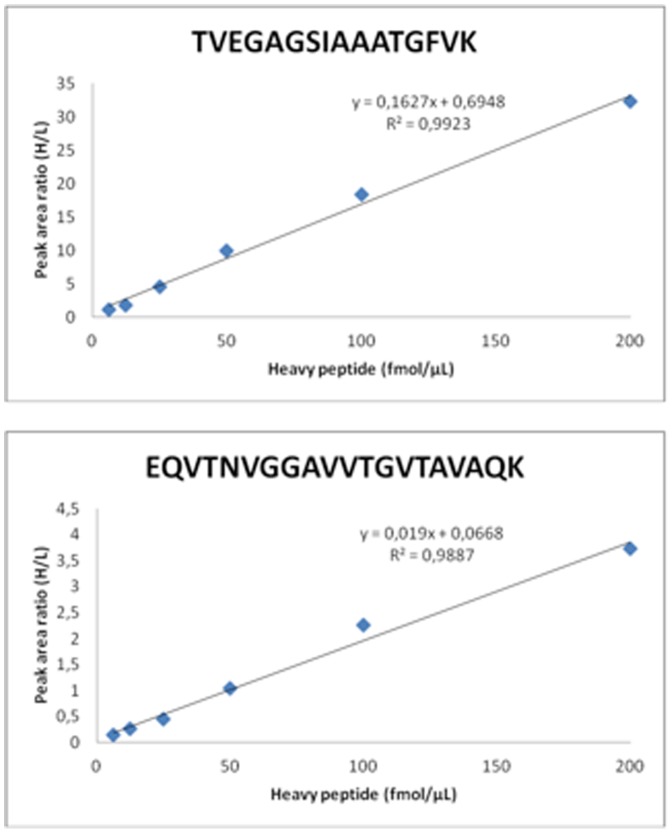
Linearity of the alpha-synuclein SRM assay determined by using heavy labeled peptides spiked into a pooled tissue sample at various estimated concentrations (6.25–200 fmol/µL).

Each analysis was repeated three times and the peak ratio of the internal standard (IS) peaks were plotted against their estimated concentrations (Figure 2). Corresponding calibration curves were generated by linear regression analysis of the peak areas (heavy/light) for both targeted peptides. The linear regression was found to be 0.99 (R^2^-values) within the investigated concentration range (6.25–200 fmol/µl).

The LOQ have been found to be 75 attomole, defined by 10 times the RSD of the noise level in the assay, and the LOD was 23 attomole, defined as three times of the noise level.

By repeated series of analysis using this SRM assay variations (RSD) were found to be 9.3% and 7.9% for TVEGAGSIAAATGFVK and EQVTNVGGAVVTGVTAVAQK, respectively.

In order to characterize the chromatographic separation of these alpha-synuclein peptides, their retention times were monitored (n = 33) with RSD values <1% for both peptides, TVEGAGSIAAATGFVK (Retention time = 19.8 min) and EQVTNVGGAVVTGVTAVAQK (Retention time = 20.5 min).

### 3.3. Haemoglobin Analysis in Tissue Samples

**Figure 3:**
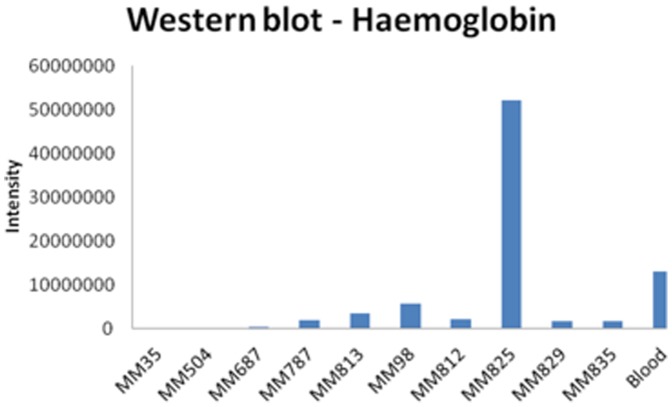
Haemoglobin expression levels in ten individual patient tissue samples and positive control (positive control corresponding to 1 µL human blood).

**Figure 4:**
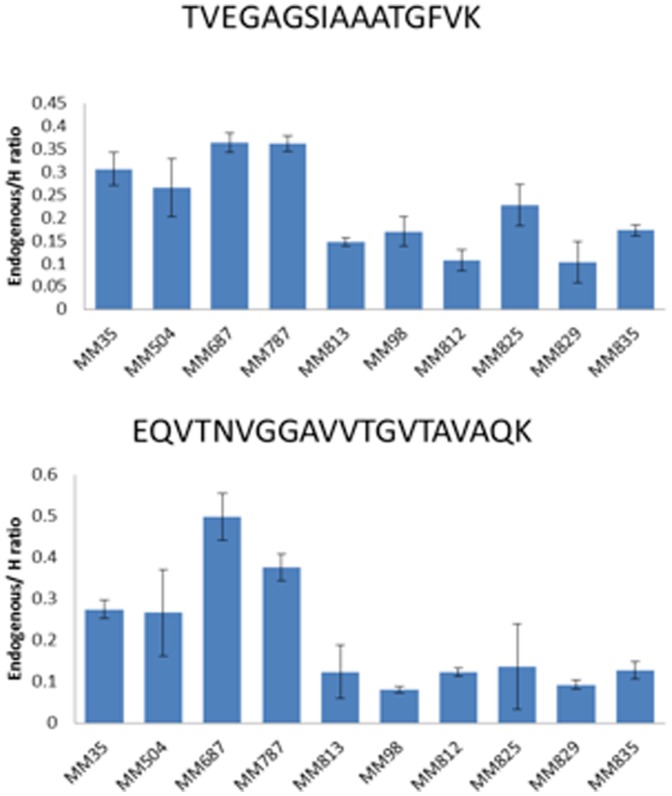
Alpha-synuclein expression levels of the two selected peptides in ten individual patient tissue samples as determined by TVEGAGSIAAATGFVK and EQVTNVGGAVVTGVTAVAQK, run in triplicates.

To understand the implication of red blood cells containing alpha-synuclein which may confound the SRM assay, we conducted haemoglobin analysis in the tumor lysates by Western blot. In most of the tumor lysates, low levels of haemoglobin were detected. Lysate from tumor MM825 seemed to contain higher levels indicating some hemolysis in that sample. Importantly, no correlation could be established between estimated levels of haemoglobin (used as a surrogate marker for red blood cells, Figure 3) and alpha-synuclein in any of the tissue samples (Figure 4), suggesting that the quantitation of alpha-synuclein in the tumor tissues by the SRM assay was not influenced by contaminating red blood cells.

### 3.4. Quantitative Expression Analysis in Biobank Tissue Samples

The tissue samples from ten MM patients were analyzed in triplicates. Reproducibility of quantification was achieved by spiking heavy isotope labeled peptides into the samples prior to LC-MS/MS analysis; resulting in a final protein amount of 1.25 µg. The amount of heavy labeled peptides was chosen in the linear range for each peptide (6.25 fmol/µL for TVEGAGSIAAATGFVK and 50 fmol/µL for EQVTNVGGAVVTGVTAVAQK). The technical validation of the alpha-synuclein assay provided evidence of low RSD values, typically <3%. The selected alpha-synuclein peptide sequences were also found to perform analytically well, with respect to transition repeatability, and could be used throughout the entire patient samples investigated in this study. RSD values by running melanoma tissues were found to be similar to those reported on earlier [47], and typically; 15–20%. Figure 4 show the results of the SRM assay performed on individual tissue samples.

**Figure 5:**
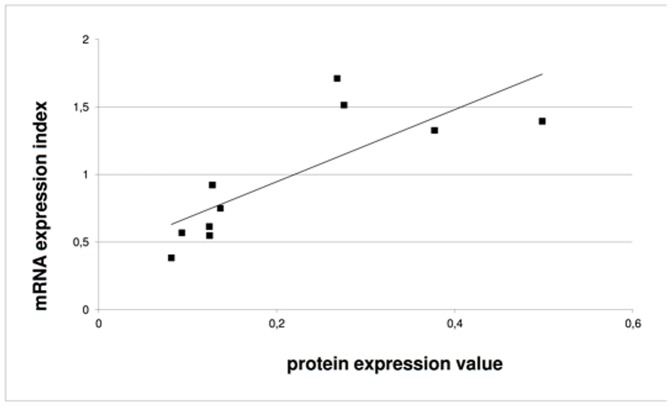
Correlation chart showing mRNA and protein expression for the 10 individual patient tissue samples. The protein expression value was calculated from the EQVTNVGGAVVTGVTAVAQK peptide as the average of total transitions. For mRNAexpression, the expression value was calculated as 2 raised to the power of expression index. Average values for triplicate measurements shown. Units are arbitrary units. Linear trend line is shown.

Four tissue samples (MM35, MM504, MM687 and MM787) showed a higher protein expression of alpha-synuclein compared to the other six tumor samples. Comparing to the mRNA expression there was a strong correlation between variation in protein levels and mRNA in the sample group (Figure 5). Correlation was indeed high, R = 0.79, and significant (p-value = 0.007).

The present data demonstrates heterogeneity of alpha-synuclein expression within MM patient lymph node metastases.

## Conclusion and Future Perspectives

We have developed and applied a SRM assay for quantification of alpha-synuclein in MM tissue lysate using a stable isotope dilution strategy. Alpha-synuclein is expressed predominantly in the brain but also in melanocytic lesions. The relationship between PD and melanoma has been observed in a number of reports, for example a comprehensive study by Gao and colleagues, where an increased risk of PD was associated with a first-degree family history of melanoma (*p* = 0.004) [13]. Along with recently published studies where a higher prevalence of developing melanoma in patients with PD was identified, this clearly supports the idea that melanoma and PD share common pathogenic pathways [12], [14], [48]. The disease relation between PD and MM indicates a possible link between functional effects of CNS disorder and cancer disease mechanisms.

Our current generic SRM assay opens up a new opportunity to quantify any alpha-synuclein form that may occur in MM, including monomer forms, truncated ones, peptides thereof, as well as oligomers, polymers and genetic variants. Specifically, the target peptides chosen in this study will enable simultaneous analysis of the four alpha-synuclein isoforms. Thus, the amount of alpha-synuclein protein detected in this study represents a summary of the four isoforms (140 kDa, 126 kDa, 112 kDa and 98 kDa). Data suggest however that specific quantification of the individual isoforms may have disease relevance and that possibly an imbalance occurs between the levels of the major form (140 kDa) and the minor forms [49] in PD. Such analysis will then require identification of new target peptides. Several general aspects for optimal selection of target peptides need to be taken into consideration [45]. Notably, these peptides should be unique to the protein to be analyzed and easily detected by mass spectrometry. When studying PTM of alpha-synuclein, adequate standards may be less accessible. However, recently chemical and semisynthetic methods to introduce sites-specific PTMs have been described [24]. Important PTM that can occur is phosphorylation at amino acid residues serine-87, serine-129, tyrosine-125, tyrosine-133 and tyrosine-136. In addition to phosphorylation, alpha-synuclein can be modified by glycosylation of serine-129 [50] and nitration at amino acid residues; tyrosine-125, tyrosine-133 and tyrosine-136 following both oxidative and nitrative stress as well as C-terminal truncation [51]. In vitro and in vivo data suggest several of these to be involved in disease pathology [3]. As a common disease presentation, somatic mutations appear in melanoma patients in both lymph nodes as well as other vital organs. These DNA mutations, resulting in amino acid shifts, have proven to be key genetic factors in cancer diseases, which can efficiently be monitored by the SRM assay format. A future mutation-SRM assay will target every single nucleotide polymorphism (SNP) that may to be considered. For melanoma, learning’s can be made from studies in PD were altered aggregation and misfolding of alpha-synuclein has been implicated. In particular mutations A53T, E46K and A60P has been linked to familial PD and may induce altered aggregation properties resulting in neurotoxicity [3]. The SRM assay format enables specific study of such mutations in tissues and body fluids by the use of specific target peptides.

Alpha-synuclein protein can be identified in various cells and compartments within melanoma patients. Vascularization and angiogenesis within melanoma is a vital part of the pathology and disease development. It is well known that in blood, erythrocytes and to minor extent platelets contain alpha-synuclein [27], [52], [53], why the origin and localization of this target protein is multifold. When developing a biomarker assay it is important to understand confounding factors like blood contamination or hemolysis effects in order for correct data interpretation. In a previous paper we presented histology images providing tissue characteristics to the tumor samples used in the present study. These images do not suggest a large variation in vascularization between the samples [54]. Furthermore, by investigating haemoglobin levels in the tumor lysates (as surrogate marker for red blood cells) no correlation could be established between haemoglobin expression and alpha-synuclein levels which verifies that the amount of alpha-synuclein detected from the tissues was not influenced by contaminating red blood cells.

The presented data demonstrates strong correlation between protein and mRNA expression levels of alpha-synuclein in metastatic tumor lysate from MM patients. With the disease relation of PD to MM this study enables investigation of the expression levels of this target protein in various forms within different MM phenotypes. The future opportunity to correlate alpha-synuclein tumor tissue levels and specific forms of the protein within different MM patient subtypes warrants further investigations.

## Supporting Information

Table S1 **SNCA mRNA levels of the ten metastases.** Gene expression was analyzed using Illumina arrays (HT12v4) and data normalized using the cubic spine method.
(DOCX)
