# Supplementary material for: Analysis of Alpha-Synuclein in Malignant Melanoma – Development of a SRM Quantification Assay
Source: PLoS One. 2014 Oct 21;9(10):e110804. doi: 10.1371/journal.pone.0110804 (PMC4204935; doi:10.1371/journal.pone.0110804)
Supplement: Table S1 — SNCA mRNA levels of the ten metastases. Gene expression was analyzed using Illumina arrays (HT12v4) and data normalized using the cubic spine method. (DOCX) [file pone.0110804.s001.docx]

| Tumor | SNCA mRNA |
| --- | --- |
| MM35  MM98  MM504  MM687  MM787  MM812  MM813  MM825  MM829  MM835 | 0.600004  -1.37733  0.775077  0.479184  0.409893  -0.69955  -0.8645  -0.41307  -0.81197  -0.11408 |

Supplementary Table 1. SNCA mRNA levels of the ten metastases. Gene expression was analyzed using Illumina arrays (HT12v4) and data normalized using the cubic spine method.
